# Cognitively Supportive Neighborhoods Slow Cognitive Decline: An 8-Year Follow-Up Among Older Chinese Americans

**DOI:** 10.1093/geroni/igaf122.2095

**Published:** 2025-12-31

**Authors:** Yanping Jiang, Wendi Da, Jessica Finlay, Fengyan Tang

**Affiliations:** Rutgers, The State University of New Jersey, New Brunswick, New Jersey, United States; Rutgers University, New Brunswick, New Jersey, United States; University of Colorado Boulder, Boulder, Colorado, United States; University of Pittsburgh, Pittsburgh, Pennsylvania, United States

## Abstract

Neighborhoods play a critical role in influencing cognitive health, particularly as most older adults prefer to age in place. Research on this topic has largely focused on composite features relevant to socioeconomic and racial composition. Specific neighborhood features, such as libraries and senior centers that can support cognitive stimulation, exercise, and social engagement, have received less attention, particularly among racially and ethnically diverse older adults. This study tests the effect of Cognability, a neighborhood index, on cognitive health in a large sample of Chinese immigrants aged 60 years and older (N = 2,787) living in metro Chicago. We computed Cognability using 13 indicators to capture cognitively supportive resources at the Census tract level using item response theory. Specifically, these13 indicators reflected availability of neighborhood resources, walkability (e.g., transit), availability of broadband internet, and availability of healthcare service. Cognitive function was assessed using various cognitive tests at baseline in 2011-2013 and every other year during 2013-2020. We included key covariates (e.g., sex, age, education) in the mixed-effects models. Results indicate that neighborhood Cognability was not associated with overall cognitive function at baseline. However, participants who lived in neighborhoods with higher Cognability scores exhibited slower cognitive decline over 8 years. This result was robust to the exclusion of participants who relocated. To conclude, our findings indicate the cognitive health benefits of living in stimulating and supportive neighborhoods in a sample of older immigrant adults. These results can inform the development of community-based interventions and policies to reduce dementia risk.

